# Deep Learning-Based Real-Time Auto Classification of Smartphone Measured Bridge Vibration Data

**DOI:** 10.3390/s20092710

**Published:** 2020-05-09

**Authors:** Ashish Shrestha, Ji Dang

**Affiliations:** 1 International Division, Civil Engineering Department, Hazama Ando Corporation, Akasaka 107-8658, Japan; 2 Department of Civil and Environmental Engineering, Saitama University, Saitama City 338-8570, Japan; dangji@mail.saitama-u.ac.jp

**Keywords:** smartphones, convolution neural network, deep learning, long-term monitoring, real-time vibration classification

## Abstract

In this study, a simple and customizable convolution neural network framework was used to train a vibration classification model that can be integrated into the measurement application in order to realize accurate and real-time bridge vibration status on mobile platforms. The inputs for the network model are basically the multichannel time-series signals acquired from the built-in accelerometer sensor of smartphones, while the outputs are the predefined vibration categories. To verify the effectiveness of the proposed framework, data collected from long-term monitoring of bridge were used for training a model, and its classification performance was evaluated on the test set constituting the data collected from the same bridge but not used previously for training. An iOS application program was developed on the smartphone for incorporating the trained model with predefined classification labels so that it can classify vibration datasets measured on any other bridges in real-time. The results justify the practical feasibility of using a low-latency, high-accuracy smartphone-based system amid which bottlenecks of processing large amounts of data will be eliminated, and stable observation of structural conditions can be promoted.

## 1. Introduction

Recent incidents such as the collapse of the I-35W Bridge in the US or the Morandi Bridge in Italy raise questions about the safety of ageing infrastructures and give a clear indication of the importance of structural health monitoring (SHM). In practice, bridge assessment includes several measures such as inspection, data interpretation, and development of engineering recommendations. Inspection as a starting point requires reliable and useful structural information on global and local performance, for which instrumentation and monitoring have been recognized as indispensable tools. Instrumentation yields vibration data, which are the basic source of information for structural dynamic analysis in the field of SHM. Obtaining information regarding structure by means of vibration data is important in disaster prevention and seismic evaluation of structures. SHM has traditionally relied on a structural identification paradigm using physics-based models where the goal is to use measurements to update a numerical (e.g., finite element) model of the structure and then deploy the model to make predictions on structural behavior. While such models are important for understanding structural behavior, such models are unlikely to be readily available for the majority of bridges. Their generation can also be resource- and time-intensive. Furthermore, computed models may not replicate the behavior of real structures due to uncertainties and approximations in the modelling process. Therefore, robust approaches for measurement interpretation that are generic and readily applicable without requiring detailed prior knowledge of structures have tremendous value in the context of extracting information from monitoring for bridge management.

Data driven approaches, which rely purely on the collected measurements for measurement interpretation, offer great promise for long term continuous monitoring. Long-term vibration measurement is one of the straightforward methods to evaluate structural integrity in terms of daily use against environmental loads, traffic loads, fatigue, etc. in addition to seismic loadings. Therefore, to incorporate long-term measurement at low cost, wireless smart sensor networks (WSSNs) are increasingly being applied in SHM around the world [1,2,3,4,5,6,7]. However, with the use of low-cost measurement systems such as WSSNs, most of the long-term vibration data inevitably contain nonstationary noise which usually occurs due to changing environmental effects and sensor-sourced faulty signals. These sensor-sourced faulty signals often occur in measured data due to hardly avoidable reasons like changing temperature and battery issues. Since these noises would significantly affect analysis results, the SHM output may be untrustworthy. Therefore, it is important to determine the normal behavior of undamaged structures as well and obtain qualitative conclusions from the changes in this behavior. Characterizing environmental effects in the measured behavior of structures and making sense of collected data still remains a significant challenge. Moreover, for post-earthquake bridge status and immediate occupation function evaluation, environmental vibration and device noise will trigger the false threshold alarm too often during seismic observation in practice. Therefore, time-series data blocks appropriate for analysis needs to be detected manually, which is often a tedious and time-consuming process that hinders the real-time evaluation of measured data for structural assessment. Also, the system needs to find some automatic method to look after the raw data and realize the vibration status by labelling and make them prepare to be used. The automation of this manual process promotes high-density and long-term observation, which is one of the major concerns currently [8,9]. Therefore, by developing a method for auto realization of time-series blocks in long-term vibration records, the bottleneck of processing a large amount data is eliminated, and stable observation of structural conditions is promoted.

The problem of auto detection can be formulized as a general classification problem and can be solved using simple neural network-based architecture or a more advanced deep learning model, such as a convolution neural network (CNN). With the tens of thousands of recorded data, which will continue to grow exponentially and indefinitely during long-term measurement, combining the powerful algorithms of CNN can make these data become the most valuable asset for structural condition assessment. In recent years, convolution neural networks have led to impressive results in object recognition [10], face verification [11] and audio classification [12]. The success of CNN in computer vision [13,14,15] in recent years has had a significant impact in a number of fields, such as image classification, transport system management [16,17] and medical imaging, with applications to the problems of damage identification and automated structural inspection [18,19,20,21,22,23,24,25,26]. Nevertheless, with the great success of deep learning, there also exists a possibility to automatically learn the feature representation from time series for time series classification (TSC) as well. However, there have not been many research efforts in the area of time series to embrace deep learning approaches in a 1D framework. Some studies that implement 1D CNN include classification of electrocardiogram (ECG) [27], fault detection in high power engines [28] and human activity recognition (HAR) problems [29,30,31], etc. Similarly, in the field of structural damage detection, researchers such as [32,33,34,35,36,37,38,39,40], etc., presented their work which involved implementing time series classification.

However, to the best knowledge of the authors’ knowledge, although all these previous works utilized CNNs to offer a robust framework for data classification, they did not provide a solution to perform those tasks in real-time using low-cost consumer grade devices like smartphones. Moreover, as the network grows deeper, the model complexity also increases correspondingly, and even a relatively small network involves millions of parameters to classify the time series data. Such costly computation complexity makes the deployment of CNN models unaffordable for common PCs and mobile devices and has not been practiced in real-time vibration classification purposes at the same time. This paper, therefore, proposes a novel data driven approach to solve this challenge. Smartphones are powerful devices that have built-in sensor infrastructures to inspect multiple parameters such as acceleration, displacement, angle, force, etc. The real-time data communication and computation adds even more advanced capabilities to these devices. With remote monitoring and real-time data mining, users can solve more applications related to structural safety assessment, save inventory costs, and help prevent unplanned downtime. Therefore, realizing the advancement in the application of smartphones for SHM, these are used as a quick bridge vibration status monitoring kit in this study. For this purpose, first of all, a simple yet computationally efficient convolution neural network-based framework has been investigated. Then, the framework is integrated into a measurement application that performs a real-time classification of measured records from smartphones by exploiting relationships in sensor inputs and automatically extracting distinct features in time domain.

## 2. System Architecture

The system architecture consists of three main layers. The first layer is responsible for data preparation, including data collection and data preprocessing. The second layer is the key layer in which feature extraction is performed using a customizable convolution neural network. The last layer is the classification layer, in which the trained classifier is integrated with the iOS smartphone operating system for real-time and in-device classification. In this study, the iOS application program developed for acceleration measurement by the authors [41] was further extended to incorporate the trained model with predefined classification labels, so that it can classify the vibration dataset measured on different bridges.

### 2.1. Data Collection

**Figure 1:**
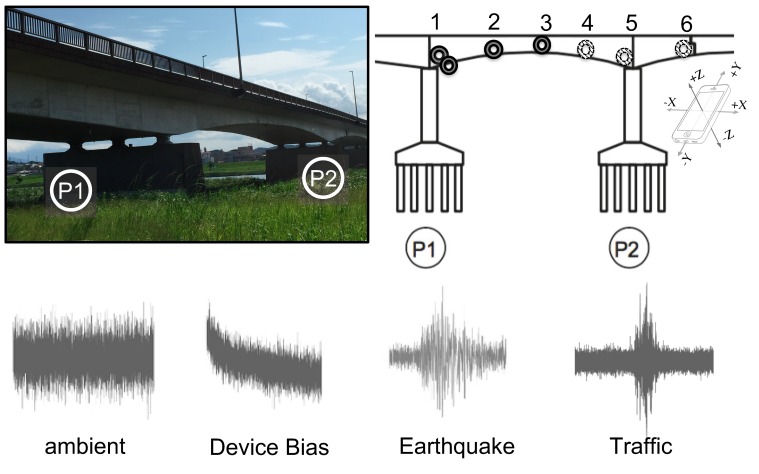
Vibration data measured at the bridge.

There currently exists no publicly available dataset of labelled time series showing different vibration categories and sensor-induced faulty signals. Thus, a new database had to be assembled from scratch to test the proposed method. Therefore, smartphones’ recorded data during long-term vibration measurement of a bridge [42] were utilized to provide the necessary resources for creating a dataset. The bridge was instrumented with six smartphones (iPhone 5s) inside of the box girder at six different locations, continually measuring acceleration vibrations as shown in Figure 1.

**Figure 2:**
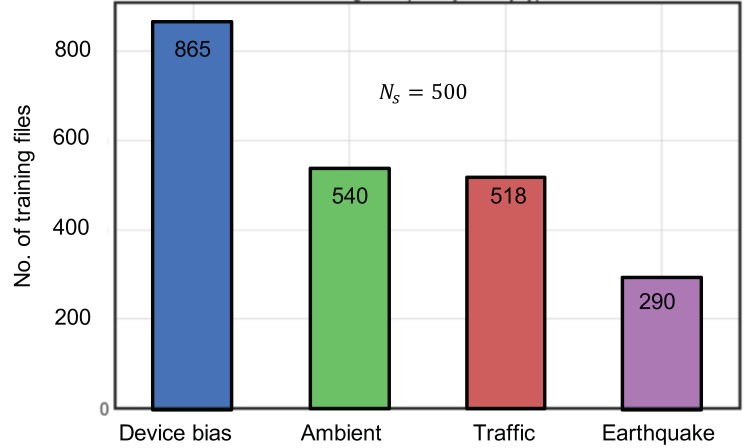
Distribution of vibration categories for training data set.

**Figure 3:**
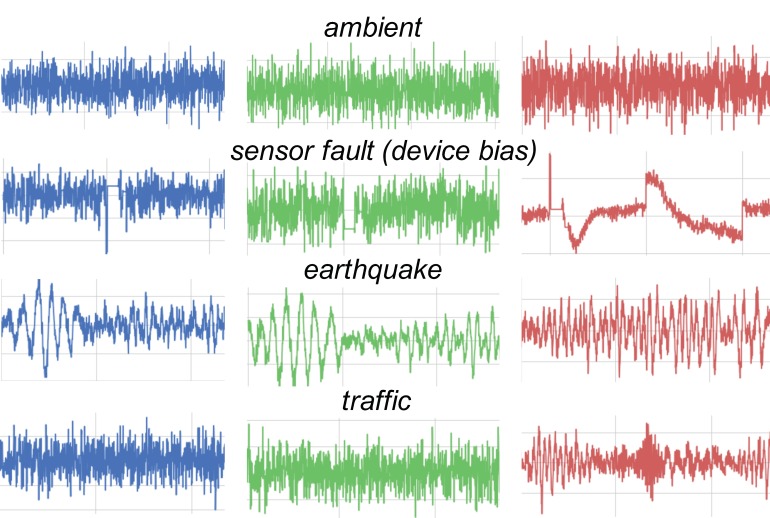
Typical waveforms illustrating different vibration types.

The vibration data collected from smartphones installed at different location of bridge mainly constitute four different types of raw acceleration data that correspond to ambient data, sensor-induced faulty data (drifts and spikes), traffic-induced vibration data, and a few earthquake records, respectively. The database includes a total of 2213 randomly selected vibration data, with each datum containing 500 sample points *N_S_*, corresponding to 5 s of acceleration record sampled at 100 Hz. All the vibration signals were hand labelled by the authors. Figure 2 shows the distribution of different types of vibration signal in the total database, while Figure 3 gives a visual illustration of different vibration types. Some common issues faced when applying the classification task are the imbalance of the dataset and the lack of enough training data. Since a real earthquake event is a rare phenomenon, it is justifiable that the earthquake class contained the least data amongst the classes.

### 2.2. Data Pre-Processing

**Figure 4:**
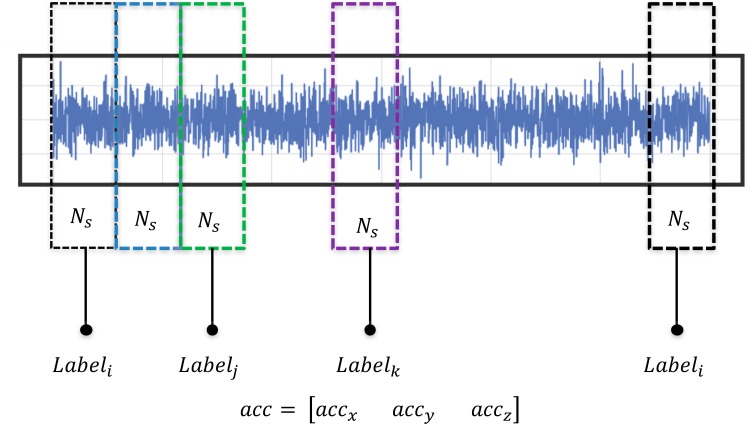
A typical time-sliced preprocessing of raw acceleration.

After creating the database, since the raw data are never available in the format that is needed for training, they need to be preprocessed. Preprocessing includes splitting the whole waveform into small segments of predefined data length $N_{S}$ and then manually assigning the appropriate label for each kind of vibration as shown in Figure 4. In this study, the data length for each sample, *N_S_*, is taken as 500. After dividing the training data into small segments, normalization is performed. Normalization with absolute maximum value amongst the training examples in a given sample is performed in this study. Similarly, since smartphones provide tri-axial acceleration measurement, the final training example for a given sample labelled with an appropriate vibration class contains acceleration values from all three axes concatenated together. This means that the final data length of a training sample is reshaped and passed into the network as a flat vector of length $3*N_{S}=1500$. 

Another preprocessing step is to separate the whole dataset into a training and a test set. The data are split in such a way that the information from the test set does not bleed into the training set. This is generally the approach for evaluating the overall performance of the model during the training and then cross-validating against the test set. The real idea behind data splitting is that the network should predict vibration characteristics from the data it has not seen before (i.e., data not used during training). Among different methods of cross-validation, K-fold cross-validation (K-fold CV) is one of the most popular methods in various applications of machine learning, especially for imbalanced classifications. It provides a less biased estimate of the performance of a model on unseen data. In order to do k-fold validation, the original sample should be first randomly partitioned into k equal sized subsets. Then, a single subsample should be selected to validate the data while the remaining k-1 subsets should be used for training. The cross-validation process should be repeated k times where each of the k subsamples is used once for validation. The obtained results shall then be averaged to produce an estimation. In this regard, for simple machine learning models such as linear regression, logistic regression, small neural networks and support vector machines which involves fewer hyperparameters, it would be better to use such a method. However, for comparatively complex models like CNNs, the K-fold cross-validation would be rather computationally expensive. Therefore, in this work, instead of using the K-fold cross-validation method, the data were randomly split into 80% and 20% for training and testing, respectively.

### 2.3. Network Architecture

In this study, a 1D CNN architecture was proposed in order to learn the feature from the raw accelerometer data obtained from smartphones. Traditionally, CNN architectures are used for image classification, which is a 2D classification problem. However, without altering the network structure, the method can also be modified for 1D classification problem by reducing the dimensionality of the convolutional layers and other hyperparameters to fit with the 1D input signals. Although many standard convolutional models such as Google Net, Alex Net, or Mobile Net are available, the advantage of utilizing a simpler model is to create a small yet highly optimized model that can be easily installed on low consumer devices like smartphones. A smaller model ensures faster computation, low memory consumption, and does not compromise the real-time classification ability of the devices.

Out of many available open source frameworks to build and train a machine learning model, in this study, “*Keras*”, a python-based machine learning framework, was implemented for the purpose of training a classifier for vibration data classification and realization. Keras provides a consistent and a simple platform for building and training models that can be trained on one backend while deploy in another. Another reason to use Keras is due to its support to integrate “*Coremltools*”, which directly creates an iOS compatible trained model that can be used in smartphone applications for real-time and in-device classification tasks [43].

**Figure 5:**
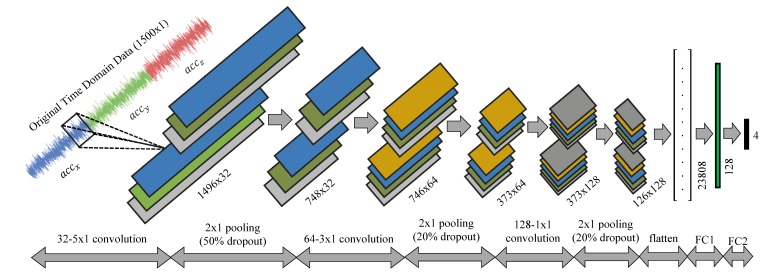
Detail map of convolution neural network (CNN) architecture for time series vibration classification.

The 1D CNN architecture adopted in this work is shown in Figure 5. It consists of three convolution layers with filter numbers of 32, 64, and 128, and sizes of 5, 3, and 1, respectively, and each is followed by a MaxPooling layer and a dropout layer with drop percentage 50%, 20% and 20%, respectively. The pooling layer halves the convolution layer’s width and height, while dropout controls the overfitting of the neurons. The use of pooling and dropout layers after each convolution layer significantly reduces the number of parameters in the fully connected layers, and training becomes faster. The lower layers use a small number of filters, while in order to process more complex parts of the input, broader filters are used. Finally, the top layers in CNN are stacked by two fully connected neural networks. These fully connected neural network are expected to combine different local structures in the lower layers for the final classification purpose. Table 1 provides a detailed overview of the CNN model.

From Table 1, it can be inferred that the total number of parameters while building a deep learning model depends on the number of variables that determine the network structure and how the network is trained. These variables are called hyperparameters. These hyperparameters are usually tweaked to find an optimum tradeoff between network accuracy and network training time. The convolution layer and pooling layer determine each layer’s output shape and number of parameters to be trained. Equations (1) and (2) define the output shape and number of parameters involved in the training, respectively.

*Output Shape = W_i_ − W_f_ + 1, H_i_ − H_f_ + 1, N_f_* (1)

No. of Parameters = N_f_ * (W_f_ * H_f_ * D_i_ + 1) (2)

where *W_i_*: input length; *W_f_*: length of filter; *H_i_*: input height; *H_f_*: height of filter; *N_f_*: filter number; and *D_i_*: input depth.

The weights from the convolutional layers are flattened and then go to the fully connected layer. The output shape from the last convolutional layer and before the fully connected layer is (186, 1, 128). Therefore, the result of flattening is an array with 23,808 elements as an input to the first fully connected layer. The first fully connected layer has 128 neurons, which means that every neuron interacts with 23,808 elements to produce a 128-neuron output layer. Finally, the 128 neurons are passed as an input to second fully connected layer to output as many neurons as the number of classes. In this model, since there are four classes, so the final output is four classes, each holding their probability of classification. The total number of trainable parameters therefore sums up to be 3,062,788. In this study, the softmax activation function combined with the categorical cross entropy loss function is used to calculate the loss as a function of difference between the true measure and predicted measure, while Stochastic Gradient Descent (SGD) algorithm minimizes the loss function. Batch size and number of epochs are arbitrarily chosen to be 200 and 30, respectively. Each epoch generally improves loss and accuracy measurement. More epochs produce a more accurate model, but training takes longer and sometimes may also lead to overfitting.

## 3. Network Accuracy and Results

**Figure 6:**
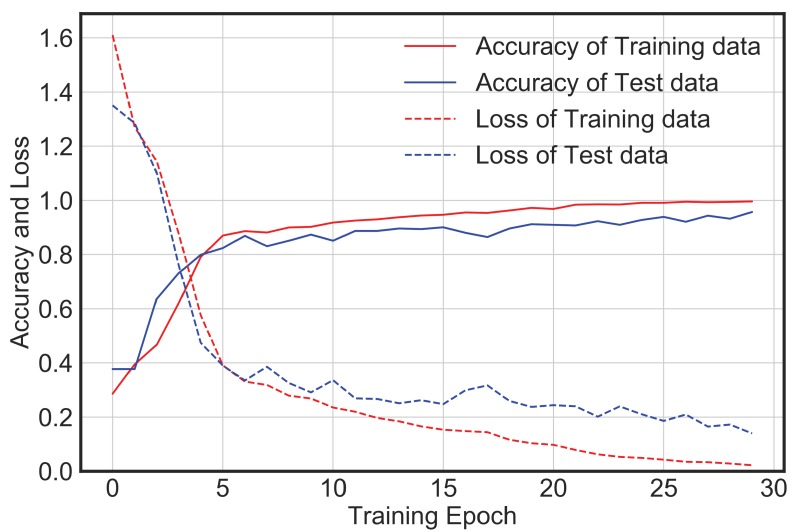
CNN model accuracy and loss for training and test dataset.

The network architecture described in the above section was implemented on the generated database to test the efficacy of its performance for the purpose of automated vibration classification. From Figure 6, it is observed that with increase in epochs, the loss value decreases, and accuracy increases. There is no significant improvement in loss or accuracy after epoch 25. Thus, overall, 30 epochs were performed. The training accuracy is around 99%, while the test accuracy is around 95%. This means that the model generalizes well for the vibration data it has not seen before.

**Figure 7:**
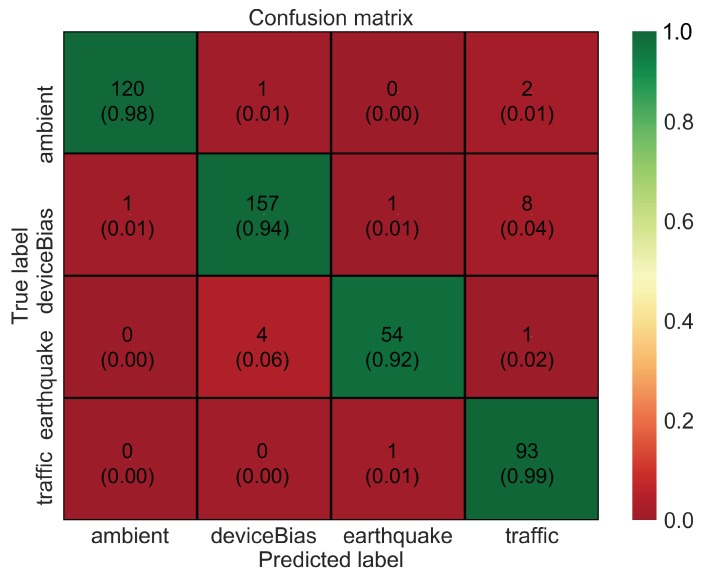
Confusion matrix for CNN model with original dataset.

**Figure 8:**
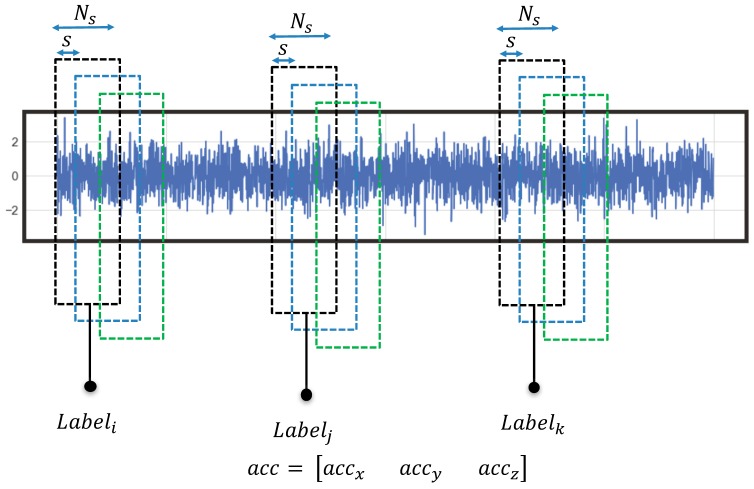
A typical time-window slicing based data augmentation.

To analyze the data in more detail, a confusion matrix is shown in Figure 7. The confusion matrix indicates that many of the prediction errors are due to confusion between three classes: bias, earthquake, and traffic. This is probably because these vibrations are relatively similar as compared to ambient vibration. Furthermore, the performance of the model based on metrics such as precision, recall, and f1-score is high, as shown in Table 2. These metrics are expressed mathematically using “Confusion Metrics”, as shown in Table 3. The overall accuracy of the model, although being high at around 95%, could still be improved further with hyperparameter tuning and especially with the data augmentation method. CNNs often tend to overfit when dealing with smaller datasets. Therefore, in this study, a time-window slicing based data augmentation method as shown in Figure 8 is proposed on the original datasets to realize more better accuracy and improvise the generalization ability.

### 3.1. Data Augmentation

As illustrated in Figure 8, let us suppose that a time series contains “N” number of data samples, length of the data chosen for classification is “N_S_”, and the time step for slicing is “s”, then this method will generate a set of $\frac{N*N_{s}}{s}$ number of sliced time series. The values of “N”, “N_S_”, and “s” adopted in this study are as follows:

“*N*” = 2213, “*N_S_*” = 500, and “*s*” = 100

Number of sliced time series = $\frac{N*N_{s}}{s}$ = $\frac{2213*500}{100}$ = 11,065

All the individual sliced time series have the same classification label as their original time series does. Therefore, by this method of selecting a suitable time-window slicing length, numerous amounts of training data can be created. Such method is especially useful whenever it is not possible to obtain enough training data for training a model. Likewise, another advantage of slicing is that the time series are not required to have equal length since we can always cut all the time series into same length using window slicing. After applying the proposed augmentation method and increasing the dataset to five times more than the original data, the CNN model is once again trained with the increased dataset while keeping the value of hyperparameters the same as before. 

**Figure 9:**
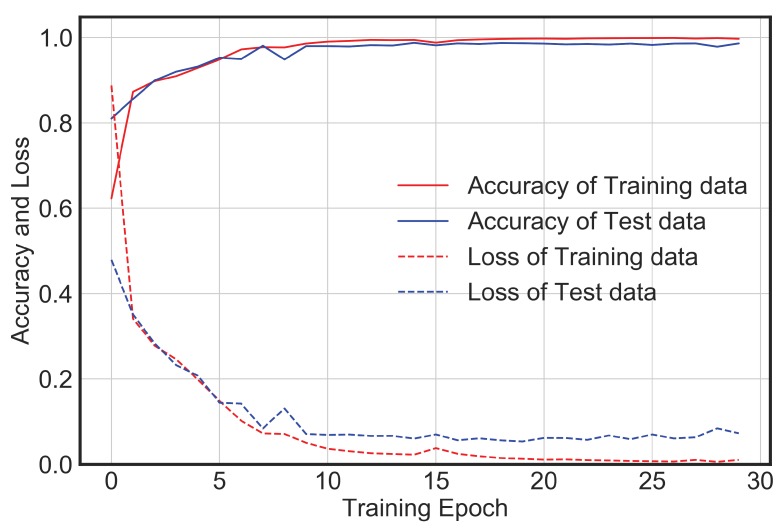
CNN model accuracy and loss for augmented dataset.

**Figure 10:**
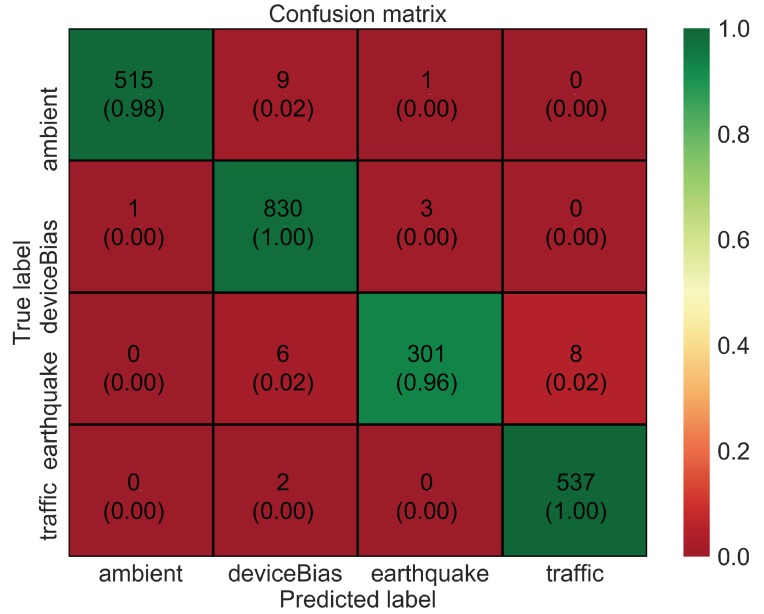
Confusion matrix for CNN model with augmented dataset.

Figure 9 shows the training accuracy and loss for the augmented dataset. With an increase in epochs, the loss value decreases, and the accuracy increases. However, in this case the model trains very fast with no significant improvement in loss or accuracy after epoch 15. The training and test accuracy with the augmented dataset are further improved to around 99.7% and 98.6%, respectively. This means that the model accurately classifies different vibration data it has not seen before. The confusion matrix, as shown in Figure 10, indicates that the error of classification between the actual label and predicted label is insignificant. Further, the performance of the model based on metrics such as precision, recall, and f1-score is also excellent, as shown in Table 4.

### 3.2. Comparison with State-of-Art Deep CNN Models and Other 1D CNN Models

In this study, in order to train the database, two state-of-the-art deep CNN models, i.e., Alex Net [10] and VGG16 [14] has also been considered as a representative of the most commonly employed CNN architectures in use today and their popularity within the research community. While it is known that deeper networks are often better in accuracy than shallow networks [44], at the same time their computational cost is high. This often consumes huge memory footprints and can hinder real-time classification as one of the key objectives of this study. 

**Figure 11:**
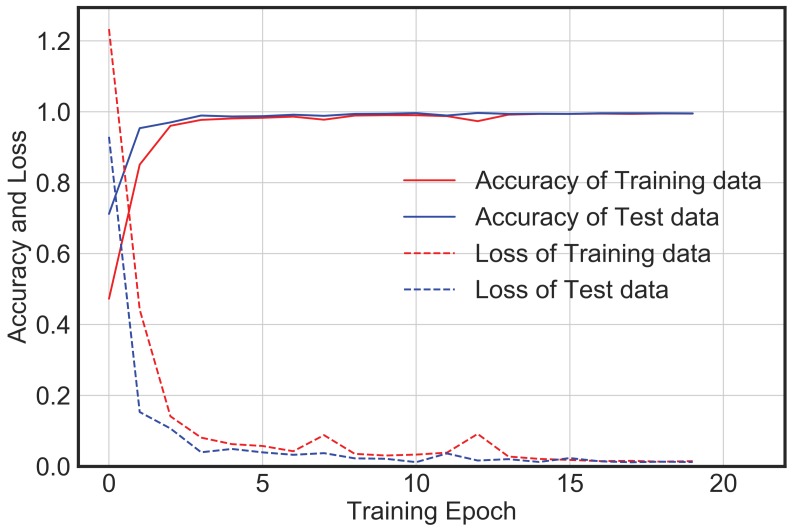
Alex Net model accuracy and loss for augmented dataset.

**Figure 12:**
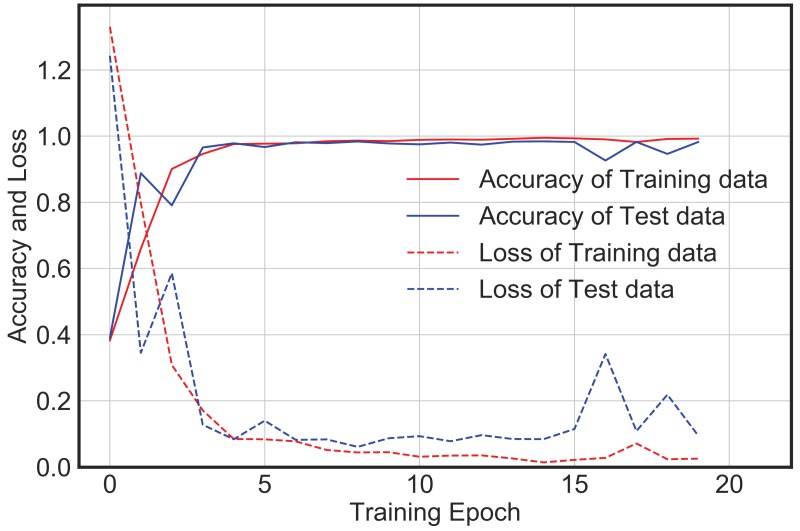
VGG16 Net model accuracy and loss for augmented dataset.

**Figure 13:**
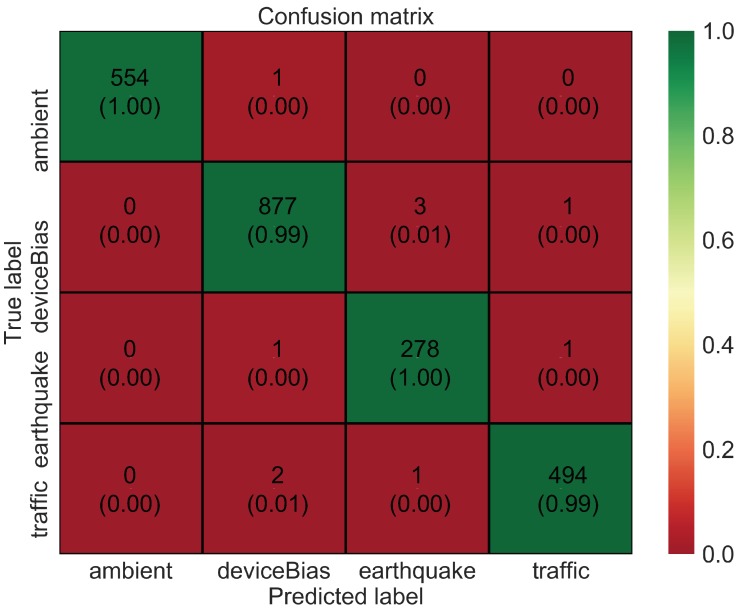
Confusion matrix for Alex Net model with augmented dataset.

**Figure 14:**
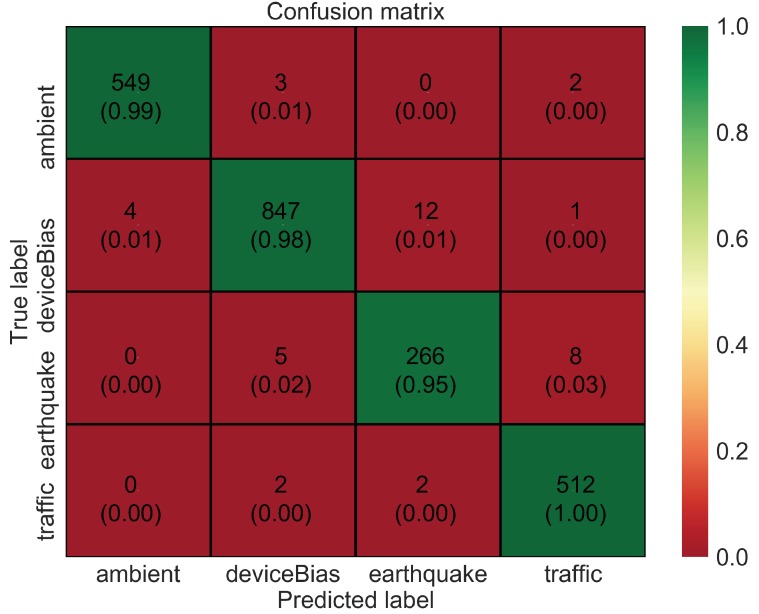
Confusion matrix for VGG16 Net model with augmented dataset.

Nevertheless, this section of the paper shows that a simple, yet computationally efficient CNN architecture as proposed in this study is also equally likely to yield similar accuracy as compared to the complex and deeper architectures for the given database and without consuming much memory. Figure 11 and Figure 12 show the training accuracy and loss for the augmented dataset using AlexNet and VGG16 Net, respectively. Both AlexNet and VGG16 are deeper networks with multiple convolutional layers followed by multiple fully connected layers. Both involve a large number of trainable parameters and take a longer time to train as compared to the proposed architecture. In both cases, the model trains very fast with no significant improvement in loss or accuracy after epoch 5. However, the final test accuracy and loss after 20 epochs for these two deeper networks as well as the proposed network is not significantly different, and the confusion matrix as shown in Figure 13 and Figure 14 indicates that the error of classification between the actual label and predicted label is also similar to that obtained from the proposed network.

**Figure 15:**
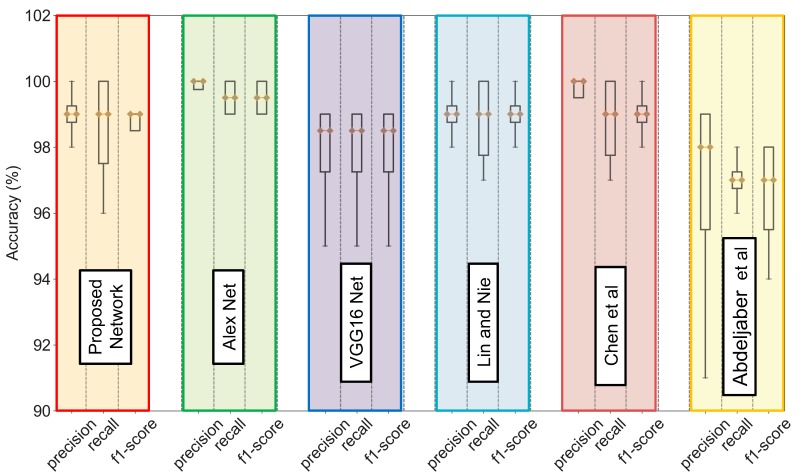
Performance comparison of different models based on precision, recall, and f1-score.

Similarly, in order to show the effectiveness of the proposed method, the performance comparison with other models involving 1D CNN, as shown in Table 5, has also been investigated. From Figure 15, it can be seen that the performance of the proposed model based on metrics such as precision, recall, and f1-score is not so significantly different from that of AlexNet and VGG16 Net and also other 1D CNN models available in the literature.

Apparently, when these trained models are finally converted to an iOS compatible format (CoreML format), there is significant difference in the memory size of these models, as listed in Table 6. The larger memory size of the trained model for deeper networks makes the computation costly and, at the same time, the deployment of such models is unaffordable for common edge devices like smartphones. Meanwhile, smaller memory size of the proposed network speeds up the computation, and their deployment in smartphones is also feasible, which allows for real-time vibration classification.

## 4. Real-Time Auto Classification of Records in Smartphones

**Figure 16:**
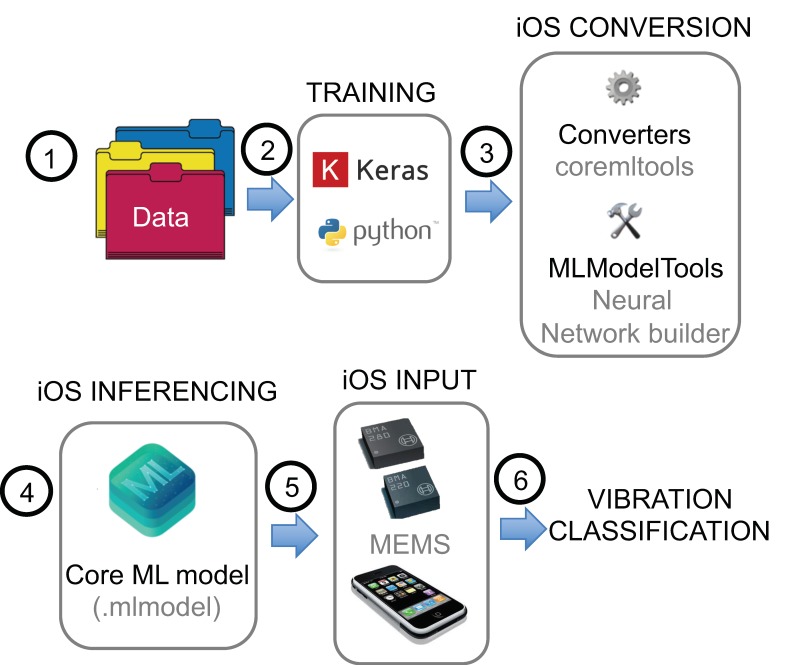
Core ML integration into smartphones for real-time vibration classification.

In this study, for realizing end-to-end processing from raw observation data to analysis result, a framework for real-time auto classification of smartphone recorded bridge vibration signals was developed following the steps as illustrated in Figure 16. The iOS application program developed for acceleration measurement [41] was further extended to incorporate the trained model with predefined classification labels, so that it can classify vibration dataset measured on any other bridges in real-time. The integration of powerful machine learning models into Apps on iOS devices is possible due to Apple’s straight forward machine learning framework known as “*Core ML*” (Core ML Apple developers [45]). The trained model as described in a previous section is converted to an iOS compatible model, which is in Core ML format, by utilizing Keras’ support for “*Coremltools*”.

Core ML is the machine learning framework used across apple products, i.e., iOS, macOS, watchOS, and tvOS. Core ML delivers fast performance with easy integration of trained machine learning models. The machine learning prediction is calculated on the device itself, due to which real-time classification performance is achievable. Core ML is optimized for on-device performance, which minimizes the memory footprint and power consumption.

There are basically three advantages of using Core ML as the machine learning on the device:

1. Low Latency and near Real-Time Results: No need to make a network API call and wait for the response. This means that such a framework is beneficial for applications such as processing the videos on successive frames.
2. Offline availability: The application runs without network connection.
3. Cost: No network connection, no API cost, and no model stored in the cloud.

However, there are some disadvantages as well. By adding the model to the device, the size of the app increases and creating an accurate model can sometimes be huge. Prediction and inference on the mobile devices involve lot of computation, which increases battery power usage and some of the older devices may have difficulties in performance. The model on the device will need to be continually trained in most cases and any change to the model results in the app needing to be updated on the device.

**Figure 17:**
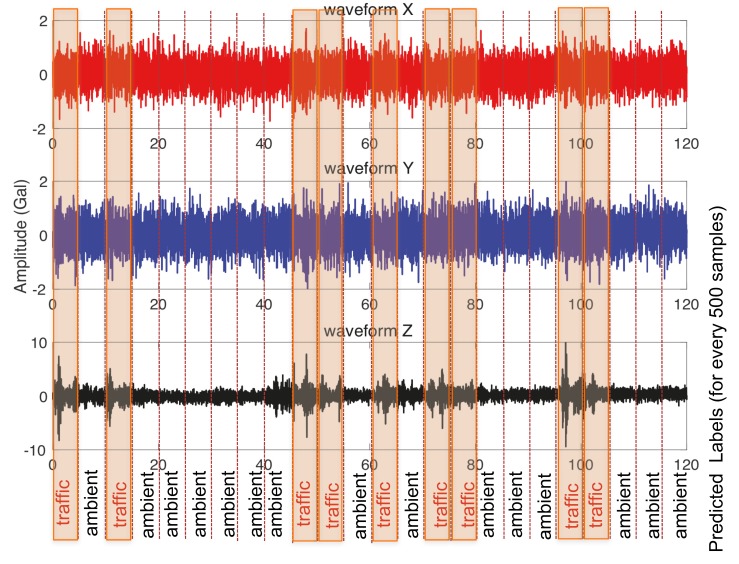
In-device prediction (classification) of vibration data.

The Core ML integrated iOS application program that measures device acceleration was then applied to verify the proposed method by collecting vibration data from random bridges. A sample example of a random three-axis acceleration measurement on bridge using smartphones for a period of 120 s and its predicted classification is shown in Figure 17. Since the inputs for the trained CNN model are the multichannel time series signals each of 500 data points acquired from a built-in accelerometer, it can be seen that the classifier predicts vibration categories (labels) to each of the 500 data points along the whole data set. Among the four pre-defined labels in the trained classifier, two of the categories (i.e., ambient and traffic) are correctly predicted. The portion of the record with significant peaks in the vertical direction corresponds to influence of traffic, while that without peaks corresponds to just ambient vibration, and the classifier predicts the classification accordingly. In this sample record, there are neither any faulty records nor an earthquake record, which therefore are not predicted by the classifier. These results demonstrate the accuracy and viability of autonomous bridge vibration realization using smartphones.

## 5. Case Study for Practical Application: Using Smartphone Measured Field Data

**Figure 18:**
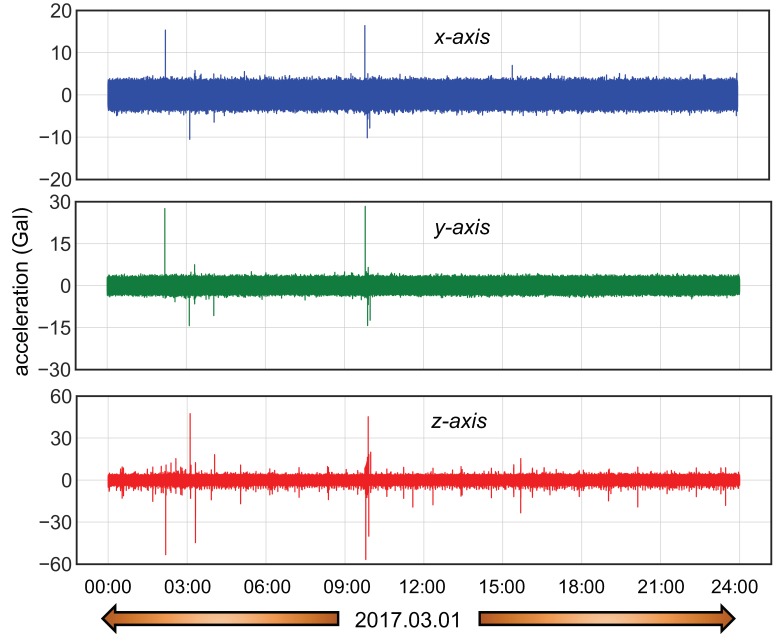
Smartphone measured field acceleration for 24 h.

Long-term vibration measurement data carried on a bridge as described in Section 2.1 were used to evaluate the performance of the proposed method in terms of robustness and accuracy in vibration classification and also utilizing the classified data to realize the status of vibration response. As a case study, one day vibration data measured at the bridge at location 3 and as shown in Figure 18 was randomly selected for the study. 

A naked eye observation of this vibration response data plot shows that along with the ambient noise, there are several small as well as large peaks which may be a result of regular traffic response, an earthquake response, or simply some non-stationary noises. Generating real-time insights out of such heterogeneous data with regard to their expanding volume is a big challenge, and this is exactly where the proposed system finds its practical applicability. Along with continuous vibration measurement, the system predicts the type of measured data (label) for every *N_s_* data points (500 data points corresponding to 5 s measurement in this study) from the trained model that is integrated into the measurement application. In addition, it also stores the vibration amplitude corresponding to the predicted label. This means that the system is capable of reporting both the vibration type as well as its intensity for every $N_{s}$ data point it measures. This prediction is done by the smartphone system in near real-time. However, the time consumption greatly depends on the CPU performance of different smartphones as shown in Table 7. Nevertheless, from Table 7 it can be seen that the time required to predict the vibration labels in-between consecutive measurements is very small, and since measurement and prediction at the same time are handled in different CPU threads, the latency in data measurement is not significant.

**Figure 19:**
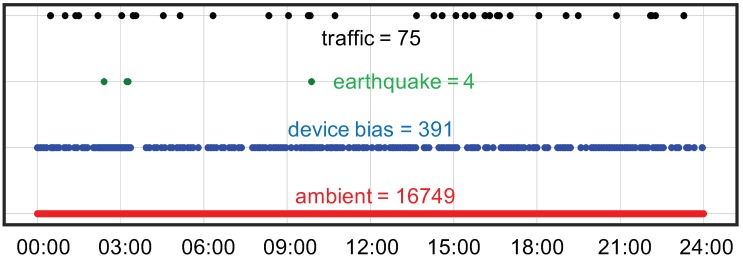
In-device prediction (classification) of measured field vibration data for 24 h.

**Figure 20:**
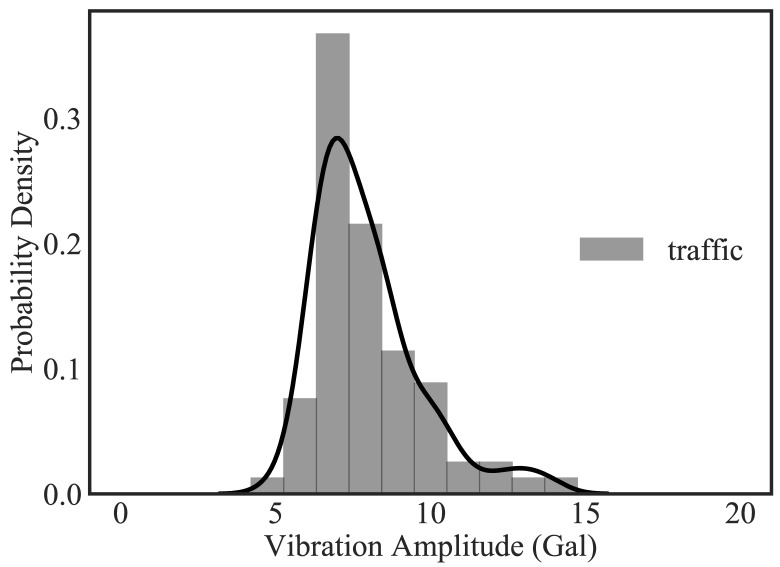
Probability density function plot of vibration amplitude for predicted traffic records.

**Figure 21:**
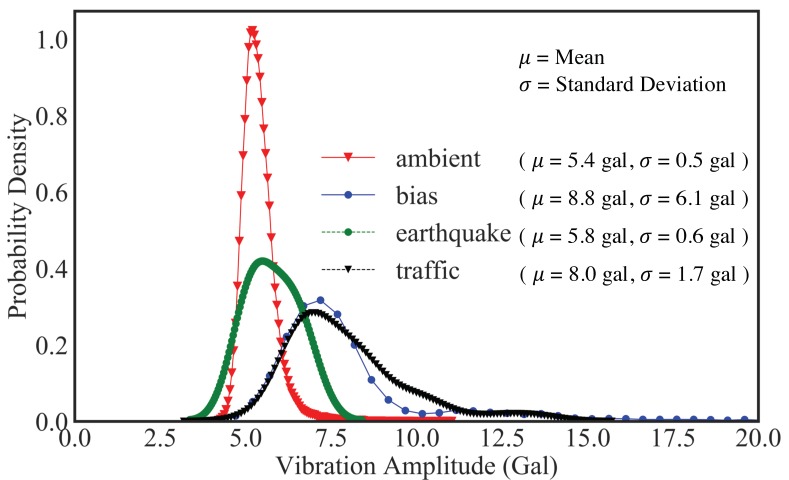
Probability density function plot of vibration amplitude for different classes of predicted records.

Figure 19 illustrates the in-device classification result of the measured field vibration data plotted in Figure 18 for a period of 24 h, while Figure 20 and Figure 21 correspond to the probability density plot of vibration amplitude for predicted traffic records and all different classes of predicted records respectively. Within this period, it can be observed that the ambient vibration constitutes thye maximum percentage of total measured vibration, with each chunk containing $N_{s}$ data points and with probability density curve scattering between 5 and 6 gal. Similarly, significant number of data chunks are predicted as traffic response and has a range of vibration amplitudes scattered between 5 and 15 gal, with 7 gal as the most frequent traffic amplitude response. Likewise, the prediction of device bias is also significant in number, with large scattering of vibration amplitude range, as depicted by the larger value of standard deviation. Since bias signals are more of a characteristic of environmental effects on sensor measurement rather than structural response, these can now be automatically filtered as not useful data during the prediction. Therefore, in this way, in addition to characterizing structural response behavior, the system also ensures that only appropriate time-series data blocks are filtered in real-time. By observing the distribution of real vibration amplitudes, users can then set a range of accurate and stable vibration safety threshold as a measure of normal or abnormal structural behavior. This method of machine monitoring thus greatly promotes the real-time evaluation of measured data for structural assessment.

In contempt of the useful application of the proposed system, it can also be seen that there are some false positive results in the form of earthquake prediction. Such deviation from the ground truth can often trigger false earthquake alarms, which reduces the performance and practical applicability of the system. Nevertheless, it can be expected that classification performance of the system can be further enhanced by increasing the training dataset, especially for earthquakes. Also, since the real earthquake data needed to train the network are rarely available, the authors will investigate the possibility of training the network based on simulated earthquake data together with the real data as a part of the future work.

## 6. Conclusions

In this study, a framework for real-time autonomous bridge vibration realization using smartphones has been investigated. The framework integrates convolution neural networks to exploit different types of relationships in sensor inputs, and automatically extract robust and distinct features in the time domain to effectively carry out classification tasks in the smartphone itself. The effectiveness of the CNN framework was verified with data collected from long-term monitoring, with results showing an excellent classification accuracy. Practical considerations and limitations of the proposed system including size of the classification app for complex and more accurate model, computational footprints, and performance variations with different smartphones was discussed.

When discussing the results on a more general level, the following conclusions can be drawn:

1. This study uses the real-world data set acquired through 24/7 data measurement in a real bridge using smartphones and utilizes those real-world data to build a real-time vibration classifier. Generally, finding a readily available real measurement data set for training the vibration classifier is a challenge in this field.
2. The overall accuracy of the model for the original dataset was improved by increasing its number using the time-window slicing-based data augmentation method.
3. The performance of the proposed model was evaluated based on confusion metrics such as precision, recall, and f1-score. It was observed that there is no significant difference in the performance results given by the proposed model as compared to state-of-art deeper networks like AlexNet and VGG16 Net, and also some other 1D CNN models applied in the literature.
4. While it is known that deeper networks are often better in accuracy than shallow networks, at the same time their computational cost is high. This often consumes huge memory footprints and can hinder real-time classification. This study shows that a simple CNN architecture, as proposed in this study, shall be the optimum model (considering both the accuracy and low memory footprint) that needs to be integrated into low computing devices such as smartphones for real-time classification purpose.
5. In this study, the classification performance of the trained model has been statistically evaluated for the test set constituting the data collected from the same bridge not used previously for training. However, similar statistical evaluation has not been presented for test on different (unseen) bridges. That would require a bunch of labelled test data collected from field measurements of different (unseen) bridges which unfortunately are not available at the time of writing this manuscript and can be considered as a part of future works. Nonetheless, the prediction classification for vibration data measured by smartphone on one of a random bridge shows that the classifier rightly predicts the vibration type. This example thus demonstrates good performance of the trained model over unseen test data and therefore justifies real-time auto classification of records in smartphones.
6. In the current study, using a simple CNN model and without much tuning of the hyperparameters, it was able to derive good classification results in short training time for the vibration database. Nonetheless, with growing network complexity, a larger database, and wider applicability of the proposed concept, adopting some more useful model tuning methods such as batch normalization and global average pooling will be studied in the future.

Historically, deep learning and machine learning have always been associated with big computers with fast CPUs, GPUs, big RAM size or running algorithms on the cloud. However, the advancement in technology has made possible the use of low-cost microcontrollers for applications in AI, due to which industries around the world are moving toward the implementation of such devices that can be readily deployed with minimal effort while providing clear advantages in cost, size, performance, power, mobility and flexibility. There are several low-cost microcontrollers which come under the base price of USD 200 or less, such as NVIDIA Jetson Nano, Coral Dev Board, Sipeed MAIX GO Suit, Raspberry Pi 4 Computer Model B, ROCK Pi 4 Model B, etc. With regard to the raw cost, performance, and power requirement, they can easily outclass those offered by the smartphones. However, in addition to the performance, getting the data to a manageable data processing and display system is important, which often requires additional supporting infrastructures such as a base station, computers, a network board, sensor nodes, batteries, software, and a gateway or a web-based monitoring software. Likewise, consideration of development time and costs, testing and certification requirements, manufacturing processes, equipment, volumes, etc. incurs additional cost which is far more expensive than building a smartphone-based system, as proposed in this work. The experience of using smartphones in this study for a machine learning paradigm provided us with valuable insights, promising guidelines, opportunities and potential of low-cost devices for structural monitoring in a rapid, remote, automated, and quantified framework. The robust implementation of such a low-cost and automated system can radically influence future advancements in smart, sustainable, and resilient infrastructure.

## Figures and Tables

**Table 1:** Detail overview of CNN model architecture used in the study.

Layer (Type)	Shape	Parameter
Conv1D (32 * 5 * 1)	1496 * 1 * 32	192
Max Pooling	748 * 1 * 32	0
Dropout (50%)	748 * 1 * 32	0
Conv1D (64 * 3 * 1)	746 * 1 * 64	6208
Max Pooling	373 * 1 * 64	0
Dropout (20%)	373 * 1 * 64	0
Conv1D (128 * 1 * 1)	373 * 1 * 128	8320
Max Pooling	186 * 1 * 128	0
Dropout (20%)	186 * 1 * 128	0
Flatten	23,808	0
Fully Connected 1	128	3,047,552
Fully Connected 2	4	516
Total Parameters: 3,062,788		

**Table 2:** Precision, recall, and f1-score for original dataset.

	Precision (P) *t_p_*/(*t_p_* + *f_p_*)	Recall (R) *t_p_*/(*t_p_* + *f_n_*)	f1-Score 2*R*P/(R + P)	No.
ambient	0.99	0.98	0.98	123
device bias	0.97	0.94	0.95	167
earthquake	0.96	0.92	0.94	59
traffic	0.89	0.99	0.94	94
avg /total	0.95	0.96	0.95	443

**Table 3:** Confusion metrics parameters.

**Predicted Class**				**The terminologies are defined as follows:** ***t_p_*: data points labeled as positive that are actually positive** ***f_p_*: data points labeled as positive that are actually negative** ***t_n_*: data points labeled as negative that are actually negative** ***f_n_*: data points labeled as negative that are actually positive**
***True Class***		**Yes**	**No**	
	**Yes**	True Positive (*t_p_*)	False Negative (*f_n_*)	
	**No**	False Positive (*f_p_*)	True Negative (*t_n_*)	

**Table 4:** Precision, recall, and f1-score for augmented dataset.

	Precision (P) *t_p_*/(*t_p_* + *f_p_*)	Recall (R) *t_p_*/(*t_p_* + *f_n_*)	f1-Score 2*R*P/(R + P)	No.
ambient	1.00	0.98	0.99	525
device bias	0.98	1.00	0.99	834
earthquake	0.99	0.96	0.97	315
traffic	0.99	1.00	0.99	539
avg/total	0.99	0.99	0.99	2213

**Table 5:** Configuration of different 1D CNN architectures [33,34,35].

Lin and Nie (2017)	Chen et al. (2018)	Abdeljaber et al. (2016)
Convo. (32 * 16 * 1)	Convo. (64 * 10 * 1)	Convo. (64 * 41 * 1)
Convo. (32 * 16 * 1)	Max. Pooling (4 * 1)	Max. Pooling (2 * 1)
Pooling (4 * 1)	Convo. (256 * 5 * 1)	Convo. (32 * 41 * 1)
Convo. (64 * 16 * 1)	Max. Pooling (4 * 1)	Max. Pooling (2 * 1)
Convo. (64 * 16 * 1)	Convo. (128 * 5 * 1)	Flatten
Pooling (4 * 1)	Max. Pooling (4 * 1)	Fully Connected (10)
Convo. (128 * 16 * 1)	Dropout (0.4)	Fully Connected (10)
Convo. (128 * 16 * 1)	Flatten	Softmax
Pooling (4 * 1)	Softmax	
Flatten		
Fully Connected (256)		
Fully Connected (128)		
Softmax		

**Table 6:** Parametric comparison with deep CNN models.

	Proposed Model	Alex Net	VGG16 Net
no. of parameters (in millions)	3.06 M	66.1 M	112 M
time for training (MacBook Pro 2.9GHz, Intel Core i5 (20 epochs)	16 min	69 min	128 min
memory size (MB)	12 MB	265 MB	450 MB

**Table 7:** Time consumption for network prediction across different smartphone models.

	iPhone 5s	iPhone 7	iPhone X
time to predict (milliseconds)	15.8	16.9	13.9
